# Relationship of α2‐Macroglobulin with Steroid‐Induced Femoral Head Necrosis: A Chinese Population‐Based Association Study in Southeast China

**DOI:** 10.1111/os.12492

**Published:** 2019-06-26

**Authors:** Shan‐hong Fang, Yong‐feng Li, Jia‐run Jiang, Peng Chen

**Affiliations:** ^1^ Department of Orthopaedics First Affiliated Hospital of Fujian Medical University Fuzhou City China; ^2^ Fujian Medical University Fuzhou City China; ^3^ Department of Orthopaedics The Hospital of Changle District Fuzhou City China

**Keywords:** α2‐Macroglobulin, Biomarkers, Microvascular vessel pathology, Steroid‐induced osteonecrosis of the femoral head

## Abstract

**Objective:**

The present study aimed to identify the relationship of α‐2‐macroglobulin and microvascular vessel pathology with steroid‐induced femoral head necrosis in the Southeast Chinese population.

**Methods:**

This study enrolled 40 patients diagnosed with steroid‐induced necrosis of the femoral head. Patients had various stages of femoral head necrosis. The differential expression of serum proteins and mRNA from patients with steroid‐induced necrosis of the femoral head (SINFH) and healthy volunteers was analyzed by western blot and quantitative polymerase chain reaction (QT‐PCR). The pathological change in osteocyte necrosis was indicated by hematoxylin and eosin stain and immunohistochemistry.

**Results:**

Hematoxylin and eosin stain showed histopathology changes in the necrotic area of patients with steroid‐induced INFH: bone trabeculae were fewer and thinner, became broken, fragmented and structurally disordered; intraosseous adipose cells became enlarged; the arrangement of the osteoblasts became irregular; and vacant bone lacunae increased. QT‐PCR showed significantly lower levels of α‐2‐macroglobulin in the serum of patients with SINFH than in controls (*P* < 0.05). Immunohistochemical staining and western blotting demonstrated that the expression of α‐2‐macroglobulin was significantly decreased in the necrotic area of SINFH patients (*P* < 0.05).

**Conclusion:**

The α‐2‐macroglobulin may be associated with the pathology of SINFH. The multiple pathological reactions occur in SINFH and α‐2‐macroglobulin may serve as a potential biomarker for the diagnosis of SINFH or a promising therapeutic target.

## Introduction

The steroid‐induced necrosis of the femoral head (SINFH) is a devastating, irreversible, and disabling disease developing following steroid therapy^1^. The functions of the hip joint are markedly impaired when the femoral head collapses. Glucocorticoid (GC) therapy is the most common cause of INFH^2^. The onset of SINFH is within several months following the administration of steroids. Due to ischemia, patients have no typical symptoms when SINFH occurs, but it is accompanied by pain and collapse of particular areas of bone. Approximately 5%–25% of patients using GC develop SINFH in the legs. Notably, in China, 53.5% of patients with severe acute respiratory syndrome who were prescribed GC developed femoral head osteonecrosis, and SINFH comprises approximately half of non‐traumatic femoral head necrosis. However, the pathogenesis of this disease remains controversial and the various theories explaining SINFH pathogenesis are predominantly classified into the osteoporosis theory, the lipid metabolism disorders theory, the vasculopathy theory, the intraosseous hypertension theory, and the GC cytotoxicity theory^3^, ^4^, ^5^, ^6^, ^7^, ^8^.

Intravascular microthrombosis or extravascular compression leads to blockage of microvessels in the femoral head and ischemic necrosis. Many studies have shown that there is a tendency of coagulation and low fibrinolysis in patients with avascular necrosis of the femoral head^9^, ^10^. The levels of membrane microparticles were significantly heightened after methylprednisolone treatment, which indicated that increased levels of membrane microparticles may be related to hypercoagulability, thrombosis, and inflammation in the microcirculation, and play an important role in SINFH^11^. Many studies have shown that α2‐macroglobulin is an important indicator for the detection of thrombotic diseases, and it plays an important role in both coagulation and fibrinolysis systems^12^. α2‐Macroglobulin has been found to enhance thrombin activity and to inhibit plasmin activity; that is, α2‐macroglobulin plays a role in regulating coagulation and fibrin hydrolysis^13^. α2‐Macroglobulin is indicated to significantly increase the regenerative capacity of plasma plasmin, while the complex formed by the binding of α2‐macroglobulin and vascular endothelial growth factor inhibits the activity of heparin, resulting in a hypercoagulable state of the blood^14^. Chen *et al*.^15^ confirmed that the level of α2‐macroglobulin in bone tissue of patients with steroid‐induced femoral head necrosis was significantly decreased, which may be related to the hypercoagulability of the blood, hyperlipidemia, free radicals, and degradation of matrix metalloproteinase. The inhibition of thrombin plays a particularly important role. A study included μCT and histological findings suggested that elevated α2M serum level is suitable as an indicative biomarker for early GC‐induced osteonecrosis of the femoral head (ONFH) in rodents, and α2M plays a role in the host reparative response to GC‐associated effects^16^.

Based on the above findings, we believe that α2‐macroglobulin plays an important role in steroid‐induced femoral head necrosis. Patients undergoing physical examination and patients with stage I–IV necrosis of the femoral head were the subjects of the present study. The levels of α2‐macroglobulin were monitored at different stages to investigate the correlation between α2‐macroglobulin and femoral head necrosis.

## Materials and Methods

### *Participants*

The study was conduct between January 2017 and March 2018 and was approved by the Institutional Review Board of The First Affiliated Hospital of Fujian Medical University. Informed consent was obtained from each patient.

Inclusion criteria: (i) participants were 20–50 years old; (ii) patients were diagnosed according to assessment by MRI; (iii) patients had received a single short course of corticosteroid medication within the 3 years prior to presentation; and (iv) control subjects were matched with patients for age and were enrolled from subjects attending routine medical checkups. The controls had no hip pain, and MRI of the hip did not show any lesions. The controls had no alcohol‐induced ONFH or other related diseases, no history of thromboembolic events, and no symptoms of hip disease.

Exclusion criteria: (i) patients with a demonstrable history of metabolic bone diseases; (ii) those who had a chronic disease history of hypertension, coronary heart disease, and diabetes; and (iii) patients younger than 20 years of age or older than 50 years of age.

After exclusion, a total of 40 patients, including 15 women and 25 men, fulfilled the inclusion and exclusion criteria. In total, 10 healthy volunteers, including 5 women and five men, were enrolled in the control group.

Patients were divided into five groups according to Association Research Circulation Osseous (ARCO) staging and no previous treatment of the femoral head. ARCO Stage 1: A band lesion of a low signal intensity around the necrotic area is seen on MRI scans. Stage 2: Subtle signs of osteosclerosis, focal osteosclerosis, or cystic change can be identified in the femoral head on MRI. Still there is no evidence of subchondral fracture. Stage 3: Early fracture in the subchondral portion is seen on MRI. Stage 4: There is osteosclerosis of the joint with accompanying joint space narrowing, acetabular changes, and destruction of the joint.

All participants signed an informed written consent prior to the study. The project was approved by the Institute's Human Research and Ethics Committees and was conducted according to the Declaration of Helsinki Principles.

### *Sample Preparation*

Peripheral venous blood (5 mL) was drawn from each patient on an empty stomach in the Outpatient Department or in the procedure room prior to general anesthesia for total hip replacement, and obtained from the 10 volunteers. The blood samples were processed to collect serum and stored at −80°C until analysis.

Blood samples (3 mL) from each subject were collected in a drying tube early in the morning and allowed to clot for 1 h at room temperature. The samples were centrifuged at 2500 *g* for 15 min at 4°C. The supernatant was dispensed into 0.5 mL aliquots and stored at −80°C until use. All the serum samples were processed according to a standard protocol. Proteins were isolated from tissues obtained from the necrotic and the normal sites of the retrieved femoral head (FH), after being cut in half at the frontal level. Tissues from the necrotic regions were obtained from the subchondral osteonecrotic area (depth 1–3 mm from cartilage) at a safe distance from the zone of regeneration, while normal tissues were obtained from the FH neck *1 cm away from the zone of regeneration.

### *Quantitative Reverse Transcription‐Polymerase Chain Reaction*

To quantitate α2‐macroglobulin mRNA transcripts, we developed and evaluated real‐time fluorescence polymerase chain reaction (PCR) assays for the Roche LightCycler (LC; Roche Diagnostics). In a separate PCR reaction, the same cDNA was evaluated for expression of the glyceraldehyde‐3‐phosphate dehydrogenase gene as the housekeeping gene. All experiments were performed in triplicate. The DNA oligonucleotide primers and the hybridization probes were synthesized by TIB Molbiol (Berlin, Germany). The adjacent ends of the hybridization probes were labeled with fluorophores.

### *Western Blot*

Western blotting experiments were performed on proteins isolated from 40 patients (necrotic and normal sites). Tissues were washed three times with ice‐cold phosphate buffered saline (PBS) and lysed with NET‐Triton Lysis Buffer. Aliquots of lysates containing 40 lg of total protein for α2‐macroglobulin detection were run on 10% NuPAGE Tris–Acetate gel (Invitrogen, Carlsbad, CA, USA) under denaturing and reducing conditions. Lysates from the osteoblastic (MC3T3‐E1) cell line were used as positive control. Immunoblot analysis was performed using rabbit monoclonal anti‐α2‐macroglobulin (Abcam, UK). Human F‐actin monoclonal antibody (Serotec, UK) was used as a protein marker for the quantification of the protein bands. Membranes were then immersed in ECL detection solution (Santa Cruz, USA) and the ratio of each α2‐macroglobulin protein band intensity relative to F‐actin band intensity was calculated for each sample.

### *Histopathology*

The pathological change of osteocyte necrosis was always indicated by the percentage of vacant bone lacunae. More specifically, five visual fields were selected and 50 bone lacunae were randomly selected from each to count vacant bone lacunae in each field via optical microscopy, at magnification ×200. These observations were used to estimate the percentage of vacant bone lacunae in the bone lacunae; that is, the vacant bone lacunae rate.

### *Immunohistochemistry*

A α2‐macroglobulin immunohistochemistry kit (Wuhan Boster Biological Technology, Fuzhou, China) was used. For the α2‐macroglobulin assay, the femoral head tissues of each group were sectioned, and streptavidin‐peroxidase immunohistochemical staining was performed to observe α2‐macroglobulin expression under a light microscope. Positive α2‐macroglobulin expression appeared as intracytoplasmic yellow particles. A known positively stained section was used as a positive control, and PBS instead of primary antibodies was used as a negative control. A total of 10 visual fields were randomly selected under a light microscope at magnification ×200, to calculate the α2‐macroglobulin‐positive cell percentage.

### *Statistical Analysis*

All the data were in the form of mean ± standard deviation, and SPSS software version 19.0 (IBM, USA) was used for statistical analysis. An independent sample *t*‐test was carried out for comparing all α2‐macroglobulin test data, and *P* < 0.01 was considered to indicate a statistically significant difference. A *t*‐test was carried out for the resulting quantitative data and in cases of heterogeneity of variance. *P* < 0.05 was considered to indicate a statistically significant difference.

## Results

### *Patient Characteristics*

The present series included 40 patients and 10 volunteers, 30 men and 20 women, with a median age of 39 years (range, 20–50 years). The patients were divided into four groups according to the ARCO four stages. There was no significant difference in the age among groups. The medical records show that all of them had received GC therapy due to arthralgia. The mean steroid dose in equivalent milligrams of prednisone was 850 mg (range, 290–3300 mg). The mean time from administration of steroids to the development of hip symptoms was 13.5 months (range, 6–26 months), but none of them fulfilled the diagnosis criteria of rheumatoid arthritis.

### *Expression of α2‐Macroglobulin is Decreased in Patients with Steroid‐Induced Osteonecrosis of the Femoral Head*

**Figure 1:**
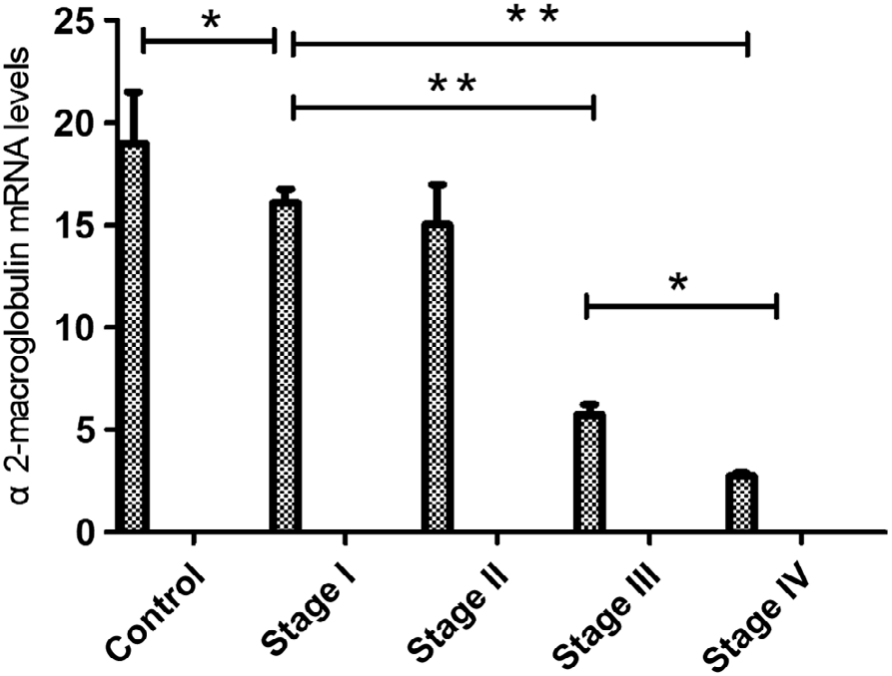
The mRNA expressions of α2‐macroglobulin in the femoral heads. The mRNA expression level of α2‐macroglobulin was significantly decreased in the patient groups compared with the control group. Compared with the stage I group, the mRNA expression levels of α2‐macroglobulin were decreased significantly in the other patient groups. *P < 0.05, **P < 0.01.

Quantitative, real‐time RT‐PCR analysis of tissues obtained from blood of 40 patients and 10 volunteers revealed a large distribution of α2‐macroglobulin mRNA expression for all 50 serum samples. The level of α2‐macroglobulin mRNA in all patients was significantly lower than in controls (*P* < 0.01, Fig. 1). As the femoral head necrosis developed, the levels of mRNA gradually decreased.

### *Histopathology Changes in the Necrotic Area of Patients with Steroid‐Induced Osteonecrosis of the Femoral Head*

**Figure 2:**
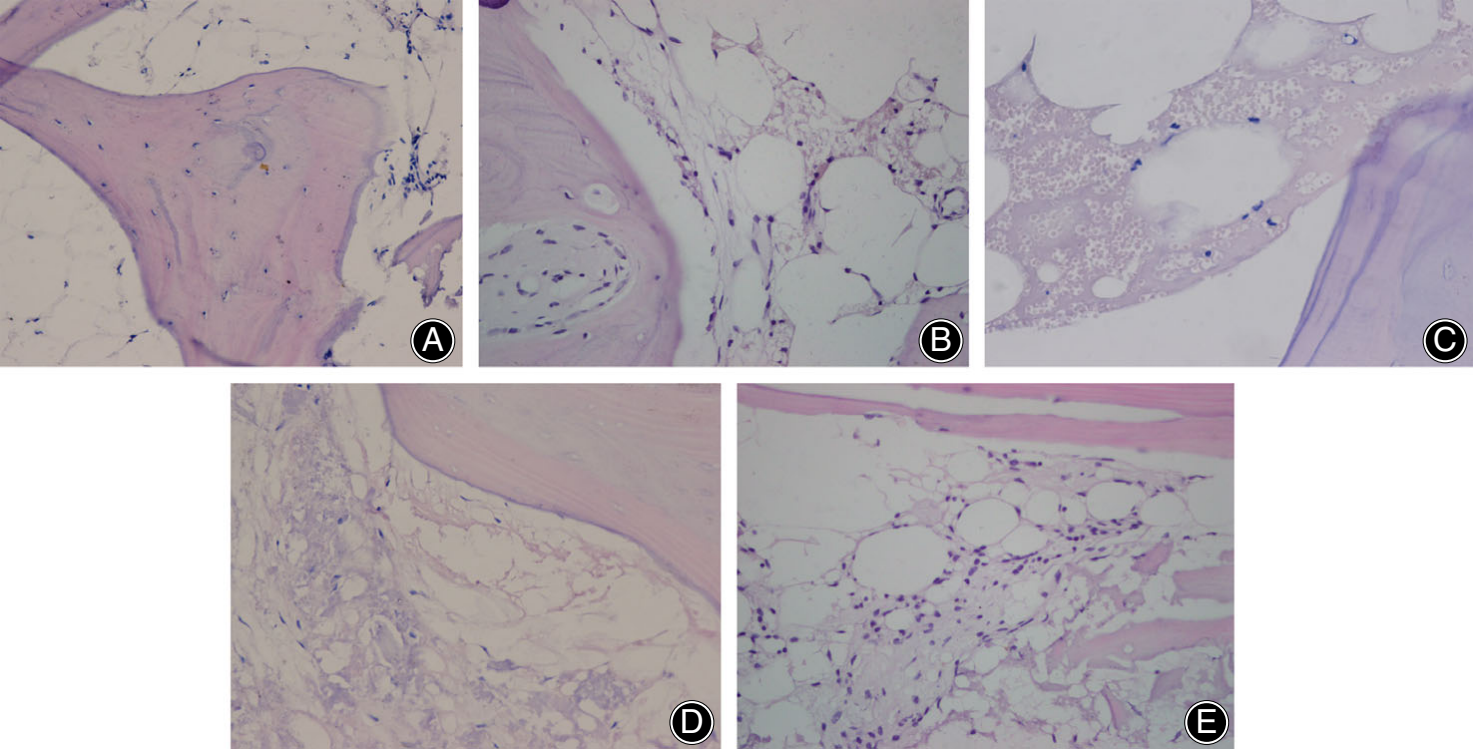
Hematoxylin–eosin staining of the femoral head necrosis (scale bar = 100 μm). The changes of necrosis were increased significantly in model groups. There was no visible necrosis in the trabeculae or bone marrow in the control group. (A) Control group; (B) stage I group; (C) stage II group; (D) stage III group; and (E) stage IV group.

Femoral head pathological sections of patient groups exhibited typical osteonecrosis; bone trabeculae were fewer and thinner, became broken, fragmented and structurally disordered; intraosseous adipose cells became enlarged; the arrangement of the osteoblasts became irregular; and vacant bone lacunae increased (Fig. 2). In the control group, the bone trabeculae remained intact, well organized, dense, and complete, and the osteocytes had big and central cell nuclei (Fig. 2A). In stage I and II groups, the bone trabeculae in the subchondral area of the femoral head were fewer and thinner, a number of bone lacunae became empty and empty bone lacunae were distributed locally; abundant hematopoietic cells existed in the medullary space and there was an increase in the number of mast adipose cells (Fig. 2B,C). In stage III and IV groups, more bone lacunae vacuoles occurred, intramedullary vessels were compressed, vascular lumens became narrow and fat embolus and thrombus were observed, bone trabeculae in the femoral head subchondral area were thin and fragile, hematopoietic cells in the medullary space became scarcer, the number of mast adipose cells increased, and the number of soft bone lacunae vacuoles increased (Fig. 2D,E).

### *Expression of α2‐Macroglobulin is Decreased in the Necrotic Area of Patients with Steroid‐Induced Osteonecrosis of the Femoral Head*

**Figure 3:**
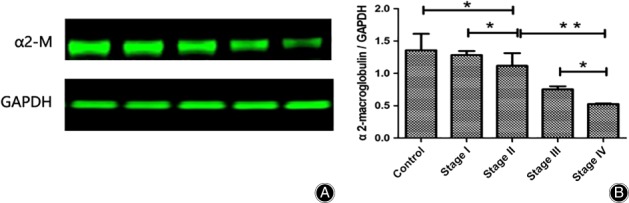
The protein levels of α2‐macroglobulin in the femoral heads. The protein expression level of α2‐macroglobulin was significantly decreased in the patient groups compared with the control group. Significant difference was noted in the protein expression levels of α2‐macroglobulin between the stage I group and the stage III group. *P < 0.05, **P < 0.01. (A) Western blots for α2‐macroglobulin expression; the bands are shown from left to right: control group; stage I group; stage II group; stage III group and; stage IV group. (B) Comparison of the α2‐macroglobulin expression among the groups.

To validate the differential expressions of α2‐macroglobulin during femoral head tissue necrosis, the proteins prepared from the necrotic femoral head tissue were analyzed by western blot. The expression of α2‐macroglobulin was significantly lower in patient groups than in the control group (Fig. 3), which is consistent with the result of mRNA levels of serum samples. However, no significant difference was observed in the levels of α2‐macroglobulin between the stage I group and the control group.

**Figure 4:**
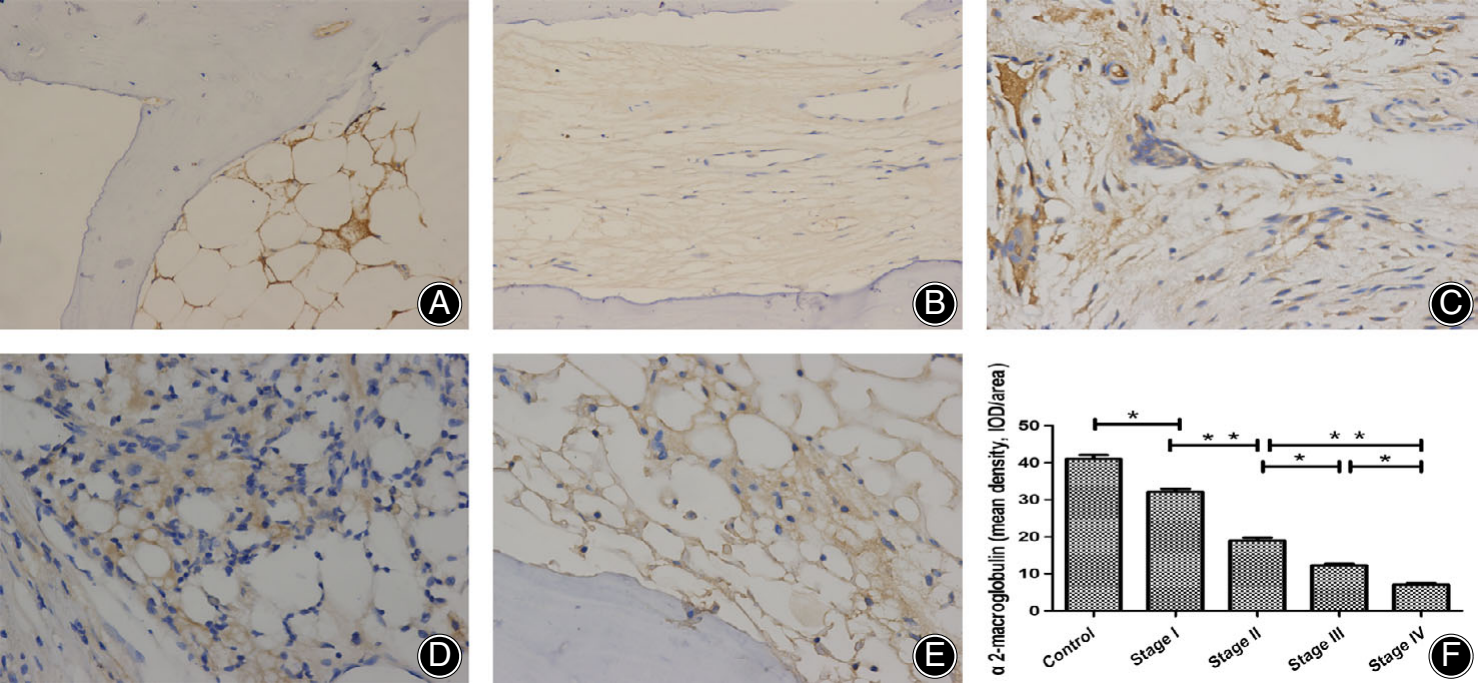
Expression of α2‐macroglobulin in the femoral heads (scale bar = 100 μm). The positive α2‐macroglobulin (brown cell) significantly increased in the patient groups compared with that in the control group. In stage III and IV groups, the positive cells increased significantly when compared with the stage I group. (A) Control group; (B) stage I group; (C) stage II group; (D) Stage III group; (E) Stage IV group. (F) Quantification of mean density of α2‐macroglobulin, P* < 0.05, P** < 0.01.

As shown in Fig. 4, there was lower expression of α2‐macroglobulin in the patient group. In the control group, brown color demonstrated more positive expression of α2‐macroglobulin. In the stage IV group, cells expressing α2‐macroglobulin were the majority; stage I and II groups were weakly positive; the stage III group was medium positive.

## Discussion

The incidence of SINFH is increasing year by year, which is mechanistically understood. It is, however, hypothesized that obstruction in blood supply and destruction of various cells such as fat cells and osteocytes produces SINFH. The obstruction in blood supply is produced by intravascular coagulation and fat emboli. Fat cell hypertrophy and osteocyte apoptosis destroy the fat cells and osteocytes. These changes result in the compromised vasculature of bone and bone marrow. This pathology leads to IBN, resulting in the failure of the mechanical strength of bone, while the diagnosis of this disorder still relies on image examination, which often fails to detect lesions in the early stage. Therefore, many patients miss the opportunities for early treatment. It is important to find the diagnostic biomarkers for SINFH.

In the present study, α2‐macroglobulin proteins showed lower expression in the serum of patients with SINFH than that of the healthy subjects. The changes were confirmed by QT‐PCR and western blotting. The expression of protein was also examined in necrotic bone tissues. α2‐macroglobulin was downregulated in the protein levels in necrotic bone tissue, consistent with the result of the serum.

The α2‐macroglobulin is an inhibitor of matrix metalloproteases (MMP)^17^, which is mainly synthesized by hepatocytes in the liver. Small amounts of α2‐macroglobulin are also produced by a number of other cells, including lung fibroblasts, macrophages, astrocytes, and tumor cells^18^, ^19^. The α2‐macroglobulin functions as a broad irreversible proteinase inhibitor and is involved in various physiological processes^20^, ^21^. The α2‐macroglobulin regulates several key factors of SINFH. The conformational change can activate α2‐macroglobulin, resulting in exposure of binding sites for its cell surface receptor, including the low‐density lipoprotein receptor‐related protein. Upon binding, α2‐macroglobulin–proteinase complexes from the extracellular matrix are rapidly removed, which blocks lipid catabolism^22^. The α2‐macroglobulin modulates blood coagulation. As reported, α2‐macroglobulin significantly enhanced plasmin generation^23^. However, α2‐macroglobulin binds vascular endothelial growth factor and the resultant α2‐macroglobulin complex inhibits heparin activity, leading to elevated coagulation. Human α2‐macroglobulin has been verified to effectively decrease the release of superoxide radicals by polynuclear leukocytes following radiation. The activity of superoxide dismutase in red cells can also be increased. The free radicals and MMP imbalance exist in the pathological process of SINFH^13^, ^24^. Along with those findings, the present study showed that α2‐macroglobulin was significantly lower in the bone tissue of patients with SINFH. Lower α2‐macroglobulin may affect the process of SINFH through these aspects. Consistent with the bone tissue, the serum α2‐macroglobulin level was also decreased.

A few limitations of this study should be mentioned here. First, this was a study with a small sample size and patients included were from a single center, which might affect both the accuracy and generalizability of these findings. No correlation analysis between these experimental indicators and stages of SINFH was performed. Thus, evidence is still weak for αM2 being a grading diagnostic indicator of SINFH. Further studies should focus on establishing a stable late stage SINFH model and further exploration of the mechanism of αM2 with SINFH. Although more studies are needed to clarify the roles of αM2 in SINFH, we believe that the results of this study will provide new clues for developing novel diagnostic indicators and preventive strategies for SINFH.

In conclusion, α2M is involved in multiple mechanisms underlying SINFH. This study underscores the critical role of α2M in the development of SINFH. Therefore, α2M may become a novel potential biomarker and a novel therapeutic target for SINFH.
